# Generating implementation evidence in clinical trials of vaccines and immunization-related technologies to reduce evidence-to-policy delays

**DOI:** 10.11604/pamj.supp.2025.51.1.47390

**Published:** 2025-07-24

**Authors:** Abdu Abdullahi Adamu, Patrick de Marie Katoto, Charles Shey Wiysonge

**Affiliations:** 1 Vaccine-Preventable Diseases Programme, World Health Organization Regional Office for Africa, Djoue, BP 06, Brazzaville, Congo,; 2 Cochrane South Africa, South African Medical Research Council, Francie van Zijl Drive, Parrow Valley, 7500, Cape Town, South Africa,; 3 Division of Epidemiology and Biostatistics, Department of Global Health, Faculty of Medicine and Health Sciences, Stellenbosch University, Cape Town, South Africa,; 4 Centre for Tropical Diseases and Global Health, Faculty of Medicine, Catholic University of Bukavu, Bukavu, Democratic Republic of Congo

**Keywords:** Immunization, implementation research, Africa

## Abstract

Evidence-to-policy delays pose a significant threat to the timely adoption of novel vaccine products despite their proven efficacy. Understanding the “how to” (i.e., implementation aspects) of innovative vaccine products can facilitate decision-making to fast-track prioritization and introduction. This perspective highlights the need to integrate implementation research within clinical trials of vaccines and immunization-related technologies to facilitate the generation of policy-relevant implementation evidence. We argue that implementation context, mechanisms, strategies, adaptation, and transportability should be systematically reported alongside efficacy outcomes to support decision-makers in informing policies regarding their use in routine settings. We propose a framework for embedding implementation research in vaccine development and advocate for stronger collaborations between research teams and policymakers. The success of implementation research within clinical trials of vaccine products depends on interdisciplinary expertise, proactive decision-maker engagement, and adherence to relevant implementation science reporting guidelines.

## Commentary

Vaccine product innovations are cardinal to preventing infectious diseases and responding to outbreaks, bolstering progress towards the Immunization Agenda 2030 (IA2030) [1,2]. The IA2030 is the global framework for immunization for this decade (2021-2030) as endorsed by the World Health Assembly [1]. The rapid advancement of vaccine innovations since the COVID-19 pandemic era has significantly reshaped the global immunization landscape, particularly in response to emerging infectious disease threats [2]. This has led to investments in targeted approaches to enhance vaccine clinical trial capabilities and capacities and explore newer vaccine platforms, especially in high-income settings [3]. Despite these advances, a persistent challenge remains: the evidence-to-policy gap, wherein efficacious vaccines face prolonged delays before policy adoption and large-scale implementation. For example, malaria and Group B Streptococcus vaccines have demonstrated efficacy yet face regulatory and policy delays [4,5]. While vaccine clinical trials traditionally focus on efficacy and safety, they often fail to generate critical implementation data that are needed for decision-making in diverse and complex health system contexts. This manuscript argues that embedding implementation research within clinical trials of vaccine products and other immunization-related technologies from the outset can generate actionable insights that accelerate the policy adoption of new vaccine products.

### Implementation science in vaccine development

Several factors precipitate evidence-to-policy delays in health systems that can affect vaccine products with proven efficacy [6]. Among these factors is the scarcity of relevant studies that elucidate the policy context of innovations, which can be addressed using implementation research [6,7]. Implementation research seeks to understand how, why, and under what conditions interventions succeed or fail in real-world settings [7]. Typically, most clinical trials of vaccine and immunization-related products primarily focus on evidence of efficacy. However, health systems are dynamic and are influenced by multiple external parameters such as institutional readiness, politics, and economics with intertwining social milieu [8]. Therefore, evidence of efficacy alone is usually insufficient to inform effective and timely decisions regarding prioritizing and introducing vaccine products. This gap can be addressed by harnessing real-world implementation evidence during clinical trials of vaccines or immunization-related products [9]. Such additional evidence that provides deeper insights into the conditions that aided the embeddedness of the vaccine or immunization-related product under development in specific health systems can benefit policymakers. Implementation evidence is classified as type 3 evidence and encompasses elements such as context, implementation strategy, implementation mechanism, adaptation, or transportability, all of which are vital for policymaking [9].

In vaccine trials, key implementation factors include context (trial setting characteristics, health systems, and regulations), implementation strategies (methods to enhance adoption), implementation mechanisms (how strategies influence outcomes), and adaptation and transportability (modifications and scalability across settings). Implementation research offers several tools that can be leveraged during vaccine clinical trials to explore these factors to aid the systematic generation of robust policy-relevant implementation evidence [7]. The field emerged in response to evidence-to-practice gaps and has been widely used to improve health service delivery in different settings [10]. When used early during product development, implementation research can unearth important evidence related to the conditions and settings under which trials of vaccine and immunization-related technologies are conducted. This way, vaccine clinical trials can simultaneously generate information on efficacy and implementation perspectives within specific contexts. Applying a policy implementation lens from the conception stage of vaccine trials can expand the evidence horizon to meet policymakers’ decision needs. Table 1 shows examples of implementation evidence that can be explored using implementation research during clinical trials of vaccine products.

**Table 1:** a structured framework for embedding implementation evidence in the context of clinical trials of vaccines and immunization-related technologies

S/No	Implementation element	Description	Examples of outcomes to be reported
1	Context	The circumstances or condition of the setting where the clinical trial of a new vaccine or immunization-related technology is being conducted.	Characteristics of the practice setting where the clinical trial was conducted (e.g., level of health care, type of health care system, human resources for health capacity and strength, ownership, source of funds) Type of incentives provided during the trial Local health policies governing the operation of the site Regulatory mechanisms Sociopolitical and economic dynamics of the setting
2	Implementation strategy	The method(s) or technique(s) for promoting the adoption, implementation, and sustainment of a new vaccine or immunization-related technology in a practice setting during clinical trials. An example of implementation strategy is training of healthcare providers in clinical trial sites.	Name of the implementation strategy Definition of the implementation strategy Description of how the implementation strategy was operationalized The actors involved in the operationalization of the implementation The action that the actors performed The target for the action performed The duration of the implementation strategy The dose or intensity of the implementation strategy The implementation outcomes that the implementation strategy affected (e.g., acceptability, adoption, penetration, fidelity, sustainment, etc.) Cost
3	Implementation mechanism	The mechanics (cause-effect relationship) of the implementation strategy used to promote the adoption, implementation, and sustainment of a new vaccine or immunization-related technology in a practice setting during clinical trials.	Causal pathway diagram, barriers to adoption
4	Adaptation	The degree to which the implementation strategies were changed or modified	The reason(s) for adaptation The process of adaption
5	Transportability	The ability to expand the clinical trial to new settings/ potential for trial findings to be applied in other settings	Penetration/ scalability, sustainability measures

* This table was adapted from a publication by Brownson et al. entitled “Revisiting concepts of evidence in implementation science” [9]

### The role of decision-maker engagement

Meaningful engagement between vaccine research teams and policymakers is essential for ensuring that trials generate implementation-relevant evidence. However, engagement levels vary, often leading to misalignment between research outputs and policy needs. A key challenge that could threaten meaningful collaboration is geographical disparity in vaccine clinical research. Most vaccine development occurs in high-income countries, whereas low- and middle-income countries, especially in Africa, bear a disproportionately higher burden of infectious diseases. To bridge this gap, decision-makers from countries where products will be deployed must be actively involved in the trial process. The approach taken by research teams, whether to engage policymakers individually or through structured platforms, also influences the effectiveness of collaboration. To address these challenges, we propose an integer-based self-rating framework (Table 2) to help research teams assess their level of decision-maker engagement in trials for vaccines and other immunization-related products. However, further research is needed to validate and standardize this framework.

**Table 2:** proposed self-rating scale for decision-maker engagement in clinical trials of vaccines and immunization-related products

Scale	Color code	Description
0		There is no engagement with country-level decision-makers on implementation evidence needs and gaps for candidate vaccines and immunization-related technologies under development.
1		There is occasional engagement with country-level decision-makers on broad issues, including implementation evidence needs and gaps for candidate vaccines and immunization-related technologies under development at either the early or late stages of clinical trials.
2		There is active involvement and participation of key decision-makers from countries to provide insights on implementation evidence needs and gaps for candidate vaccines and immunization-related technologies under development in late-stage clinical trials.
3		There is active involvement and participation of key decision-makers from countries to provide insights on implementation evidence needs and gaps for candidate vaccines and immunization-related technologies under development from early-stage clinical trials
4		There is active involvement, and participation of well-functioning, country-based, multistakeholder engagement mechanisms for identifying implementation evidence needs and gaps for candidate vaccines and immunization-related technologies under development in late-stage clinical trials
5		There is active involvement, and participation of well-functioning, country-based, multistakeholder engagement mechanisms for identifying implementation evidence needs and gaps for candidate vaccines and immunization-related technologies under development from early-stage clinical trials

### Ethical and feasibility considerations

Integrating implementation research into clinical trials introduces ethical and feasibility challenges, including informed consent, ensuring participants fully understand both efficacy and implementation objectives; data sharing and transparency, promoting open access to implementation data for reproducibility and policy impact; and conflict of interest management, structuring decision-maker involvement to prevent undue influence on trial outcomes. To maximize the benefits of implementation research in vaccine development, countries can establish national coordination platforms that facilitate collaboration between researchers and policymakers. For example, a National Technical Working Group for Vaccine Implementation Research could be established to collaborate with clinical trial teams, ensuring that research outcomes address broader implementation needs and inform timely policy decisions. Such groups should comprise representatives from the ministries of health, immunization programs, national advisory bodies, ethics committees, regulatory agencies, academic Institutions, implementation science alliances, and global immunization partners, tailored to each country’s context. Strengthening these platforms through vaccine development partnerships can enhance their capacity to drive evidence-based immunization policies and accelerate vaccine adoption.

## Conclusion

Implementation research has the potential to facilitate faster translation of new vaccine products that are known to work into policies. However, evidence-to-policy delays continue to hinder the timely adoption of novel vaccines. This manuscript advocates for embedding implementation research within vaccine trials as a practical strategy to generate policy-relevant evidence. We propose a structured framework to guide researchers in systematically documenting contextual and implementation factors. Additionally, proactive engagement of decision-makers and ethical oversight are essential to ensure that implementation data informs national immunization policies effectively. Moving forward, greater interdisciplinary collaboration, improved methodological rigor in implementation research, and stronger institutional support for decision-maker engagement are necessary to bridge the evidence-to-policy gap.
